# NovoSorb™ biodegradable temporizing matrix for post-excision wound reconstruction in a paediatric giant congenital melanocytic naevus: a case report

**DOI:** 10.1093/jscr/rjag875

**Published:** 2026-10-04

**Authors:** Kelly Ka Yee Chu, Calver Pang, Kana Miyagi

**Affiliations:** Department of Plastic and Reconstructive Surgery, Lister Hospital, East & North Hertfordshire NHS Trust, Coreys Mill Lane, Stevenage SG1 4AB, United Kingdom; Department of Plastic and Reconstructive Surgery, Addenbrooke’s Hospital, Cambridge University Hospitals NHS Foundation Trust, Hills Road, Cambridge CB2 2QQ, United Kingdom; Department of Surgical Biotechnology, Division of Surgery and Interventional Science, University College London, Gower Street, London WC1E 6BT, United Kingdom; Department of Plastic and Reconstructive Surgery, Addenbrooke’s Hospital, Cambridge University Hospitals NHS Foundation Trust, Hills Road, Cambridge CB2 2QQ, United Kingdom

**Keywords:** giant congenital melanocytic naevus, dermal substitute, biodegradable temporizing matrix, paediatric reconstruction, wound management, skin grafting

## Abstract

Giant congenital melanocytic naevi (GCMN) presenting over large surface areas pose significant reconstructive challenges following surgical excision. We report the case of a paediatric female with a symptomatic GCMN covering ~2% of total body surface area on the right thigh, causing recurrent ulceration and psychological distress. Following failed conservative management, excision under general anaesthetic was performed, with reconstruction of the thigh defect using NovoSorb™ Biodegradable Temporizing Matrix (BTM). Despite a series of sequential infectious and contamination episodes during integration—including bacterial infection with *Escherichia coli, Pseudomonas aeruginosa*, and *Klebsiella variicola*, urine contamination, and maggot infestation—BTM maintained integration with conservative management. Successful split-thickness skin grafting was achieved at 4 weeks, with complete wound healing at 4 months and a well-matured scar at 1 year. This case supports BTM as a robust option for paediatric GCMN reconstruction in anatomically challenging, high-contamination locations.

## Introduction

Giant congenital melanocytic naevi (GCMN) present significant reconstructive challenges following surgical excision, particularly when they cover large surface areas where primary closure or flap reconstruction is not feasible. While the lifetime risk of malignant transformation remains debated, estimated between 0.7% and 2.9%, surgical excision is frequently pursued for cosmetic, psychological, and oncological reasons [1, 2]. Reconstruction of resulting defects often requires dermal substitutes to supplement split-thickness skin grafting (STSG) when donor tissue is limited [3].

Several dermal templates are commercially available, including NovoSorb™ Biodegradable Temporizing Matrix (BTM), Integra®, and Matriderm®, each differing in composition, staging requirements, and infection resistance [4]. Evidence for their use in paediatric GCMN excision remains limited to small case series and reports [3, 5]. We present a case of BTM used for thigh reconstruction following GCMN excision in a child, with a focus on infection resistance and operative decision-making in a challenging anatomical location.

## Case report

A female of middle childhood (45.5 kg, 141.5 cm) was referred to our Dermatology department with a congenital naevus on the right thigh. The lesion covered ~2% of total body surface area, affecting 30%–40% of the medial thigh circumferentially and measuring 20 × 10–13 cm at presentation, with flat brown pigmentation. She had no relevant personal or family history. In the absence of high-risk features—multiple satellite naevi, posterior axial location, or head and neck involvement—magnetic resonance imaging (MRI) was not indicated [6, 7].

Over 4 years, the lesion grew progressively symptomatic: skin thickening, irregular pigmentation, and recurrent ulceration with bleeding. A biopsy was performed, confirming no malignancy. By the time of surgical planning, the GCMN measured 27 × 25 cm with minimal skin laxity, bled through clothing, and was causing significant psychological distress affecting school and social life.

Five years post-presentation, with failure of topical treatments to reduce GCMN size, the patient opted for surgical excision of the lesion under general anaesthetic. The thigh defect was resurfaced with BTM, secured with staples, quilting sutures, and 4/0 Prolene along the superior border at the groin crease. Although the lesion extended into the right perineum, reconstruction was deliberately limited to the area proximal to the groin crease. This decision reflected the prohibitive risk of faecal and urinary contamination distal to this border and the technical difficulty of maintaining an adequate vacuum-assisted closure (VAC) seal near the vulval area; the perineal portion was closed primarily. Smith & Nephew's PICO Negative Pressure Wound Therapy system dressing was trialled but could not maintain suction over the wound area, and a 3M™ ActiV.A.C.™ Therapy Unit was applied. The patient was discharged the same day with 5 days of oral co-amoxiclav. Histology confirmed excision to subcutaneous fat (up to 5 mm depth), with epidermal hyperplasia and intradermal component extending into the subcutis. No malignancy was identified.

During BTM integration, a sequence of graft contaminations and infections ensued (Table 1). In summary, malodorous turbid fluid and heavy *Escherichia coli* growth were identified at post-operative day (POD) 8 (Fig. 1); suspected urine contamination with coliform bacilli was found at POD 16 (Fig. 2); mixed gram-negative flora persisted at POD 28; maggot infestation was identified at POD 35 (Fig. 3); and heavy *Pseudomonas aeruginosa* and *Klebsiella variicola* were cultured at POD 42. Throughout this period, the BTM maintained integration, managed with four courses of oral antibiotics and six wound washes in theatre.

**Table 1:** Timeline of procedures, wound status, and microbiology.

Figure	POD	Setting	Procedures and medications	Wound status	Microbiology
	0	Theatre (GA)	GCMN excision; BTM implantation; VAC dressing; direct primary closure of perineal defect; 5 days co-amoxiclav	–	–
Fig. 1	8	Outpatient clinic	Wound swab; wound cleaned; VAC dressing changed	Well in self; no fever; manageable pain; malodorous turbid brown fluid from infero-posterior wound; BTM mostly red/pink and well-adhered	Heavy growth *E. coli*; mixed skin flora
Fig. 2	16	Theatre (GA)	Wound wash and dressing under GA due to patient distress	Most BTM integrating well; few small (<1 cm) pale areas; offensive odour consistent with urine contamination	Heavy growth coliform bacilli; mixed skin flora
	21	Outpatient clinic	Wound cleaned and redressed; Tegaderm and Aquacel applied for waterproofing; 1 week co-amoxiclav	BTM well-adhered, mostly red/pink, more yellow near wound edges; considerable malodour; visible soilage	–
	28	Outpatient clinic	Wound clean; dressing change; wound swab	Dressing wet over buttock area	Heavy growth mixed gram-negative flora; mixed skin flora
Fig. 3	35	Theatre (GA)	Wound cleaned and redressed	Maggots in dressing over BTM; BTM integrated—pale salmon pink, blanching; partial sealing membrane elevation postero-medially	Heavy growth mixed gram-negative flora; mixed skin flora
	38	Theatre (GA)	Thigh cleaned with chlorhexidine; bactigras dressing	No maggots; minimal over-granulation at BTM neo dermis margins; some sealing membrane delamination; wound visibly unclean	Heavy growth mixed gram-negative flora; light growth mixed skin flora
	42	Outpatient clinic	Wound swab	–	Heavy growth *P. aeruginosa*; *K. variicola*; anaerobic organisms
Fig. 4	44	Theatre (GA)	BTM sealing membrane removed; over-granulation debrided; 1:1.15 meshed STSG from left thigh applied with LiquiBand; VAC at −125 mmHg; 5 days co-amoxiclav	Wound macroscopically clean; 100% BTM take with blanching on pressure; over-granulation at posterior, superior, and inferior margins	–
	51	Theatre (GA)	VAC dressing reapplied; IV Tazocin on induction; 1 week ciprofloxacin	*Pseudomonas* wound odour; turbid brown fluid in canister; 90% graft take	Heavy growth *P. aeruginosa*; scant mixed skin flora
	54	Theatre (GA)	Bilateral thigh wound wash, clean, and dressing	80% graft take; minimal over-granulation through mesh; minimal canister fluid	Heavy growth mixed gram-negative flora; moderate growth *P. aeruginosa*; scant mixed skin flora
Fig. 5	65	Theatre (GA)	Fucidin H and bactigras to over-granulation; Octenisan soap and Aquamax emollient supplied; wound hygiene and mobilization advice	Spots of eschar; some over-granulation	–
	10 weeks	Outpatient clinic	Wound cleaned; Fucidin H and bactigras to over-granulation	Largely healed; no signs of infection	–
Figs 6 and 7	4 months	Outpatient clinic	General follow-up	Three small (~1.5 cm) epithelializing areas remaining; wound otherwise dry; good contour; scar colour fading; patient returning to school	–
Figs 8–10	1 year	Outpatient clinic	General follow-up; 1-year review	Fully healed maturing scar; colour fading; possible hypertrophic scar at graft seam	–

Fig. = Figure; POD = post-operative day; BTM = biodegradable temporising matrix; STSG = split-thickness skin graft; GA = general anaesthetic; VAC = vacuum-assisted closure.

**Figure 1:**
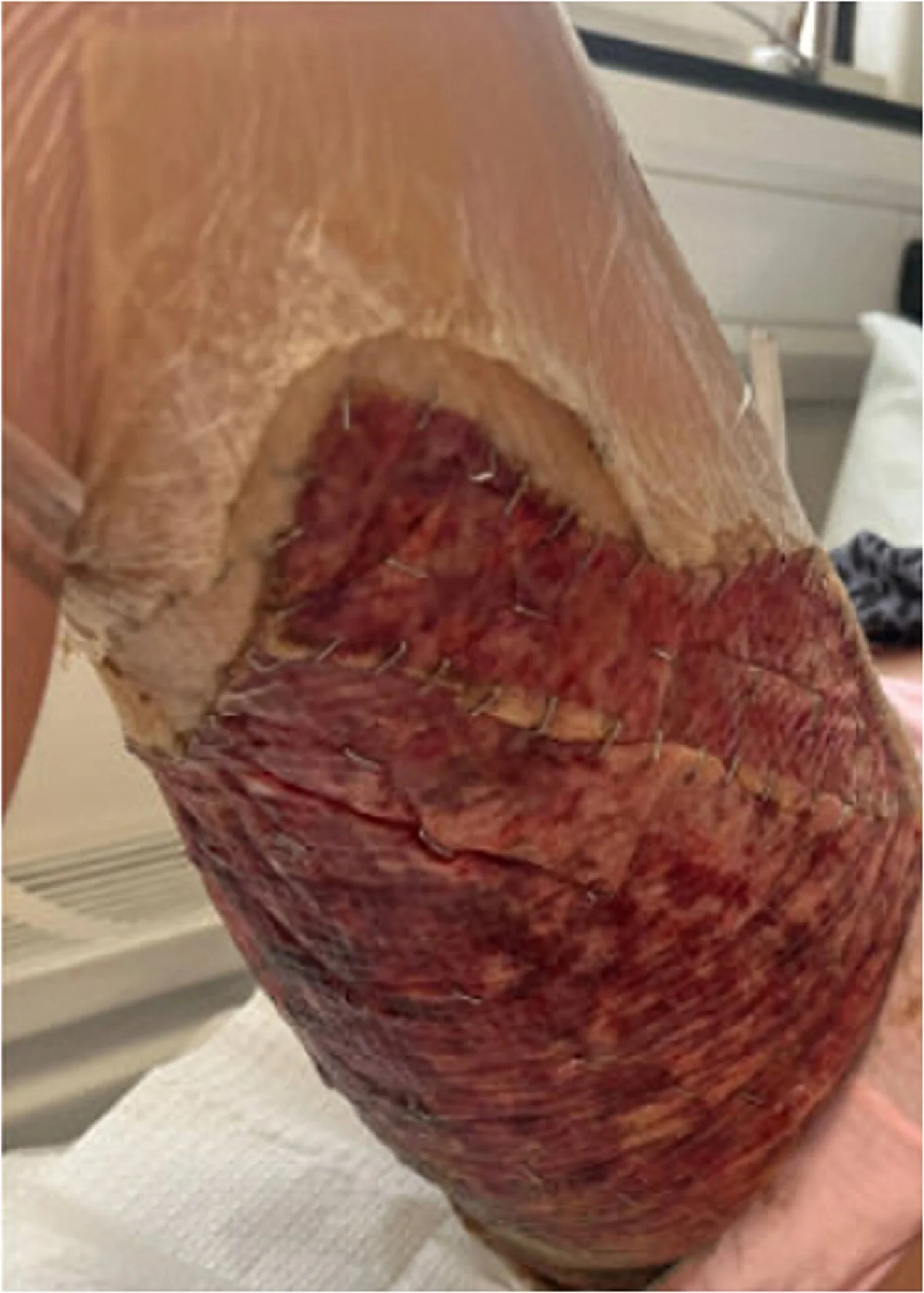
Medial thigh wound at POD 8 showing well-adhered BTM with malodorous turbid fluid at the wound margin.

**Figure 2:**
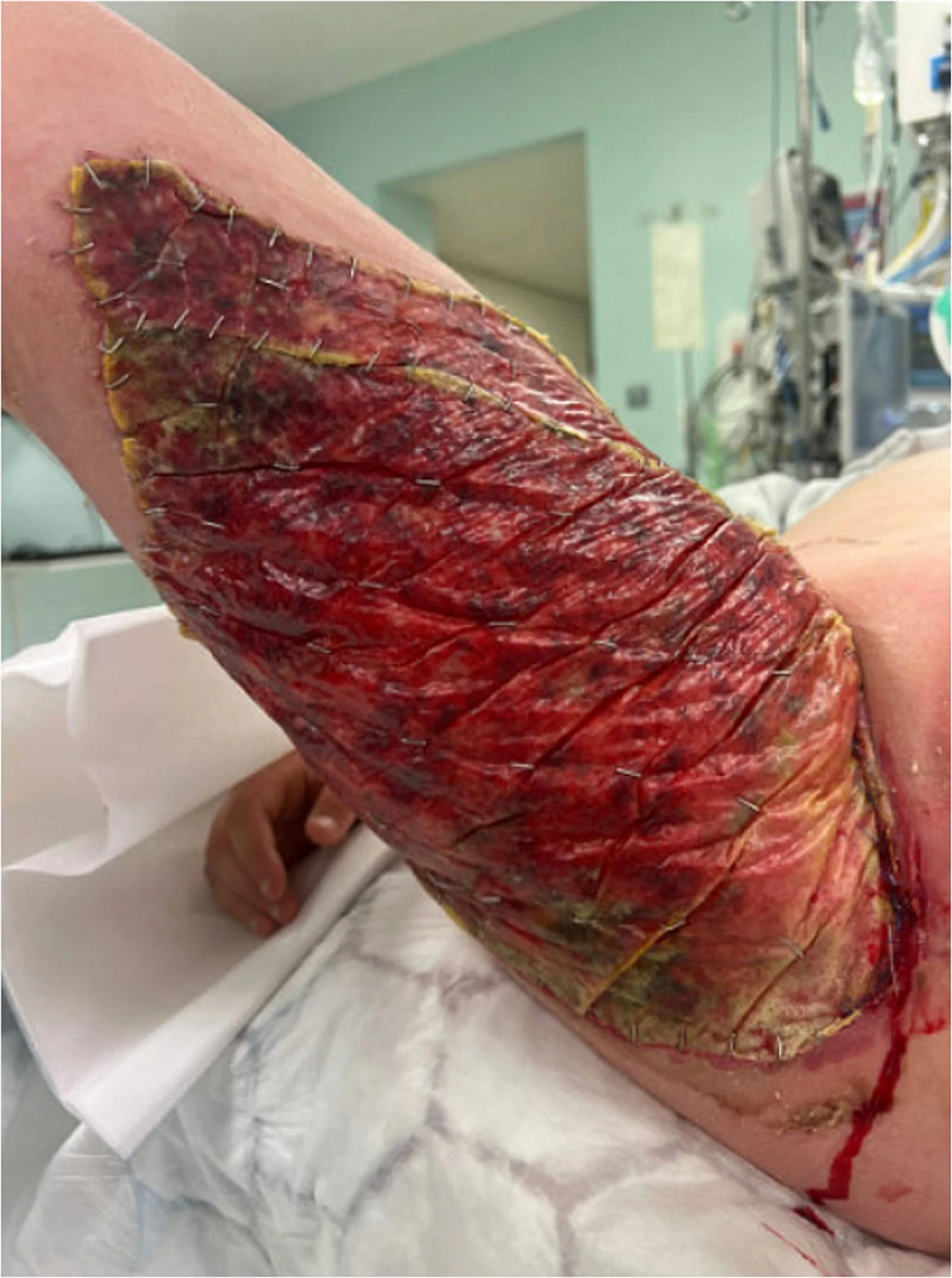
Medial thigh wound at POD 16 showing BTM integrating with predominantly red/pink appearance and small pale areas at the periphery.

**Figure 3:**
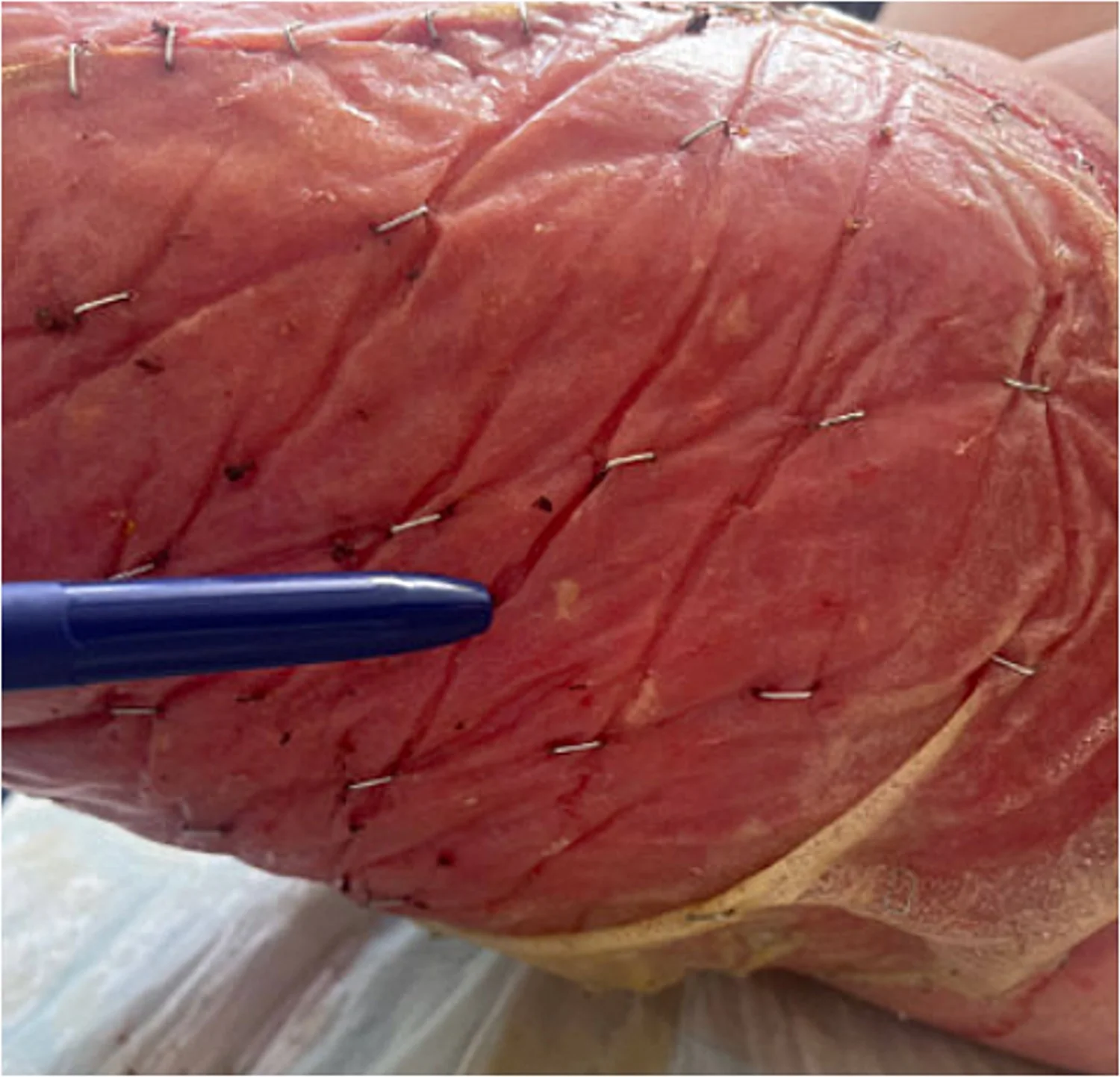
Medial thigh wound at POD 35 showing maggot infestation overlying integrated BTM neo dermis.

At POD 44, 100% BTM take was confirmed macroscopically (Fig. 4); the sealing membrane was removed, over-granulation at wound margins debrided, and a 1:1.15 meshed STSG harvested from the left thigh was applied and secured with LiquiBand glue, with VAC dressing applied at −125 mmHg. A further 5 days of co-amoxiclav were prescribed. By POD 54, 80% STSG take was observed with minimal over-granulation through the mesh. Wound healing was otherwise progressive, with no further significant complications (Fig. 5).

**Figure 4:**
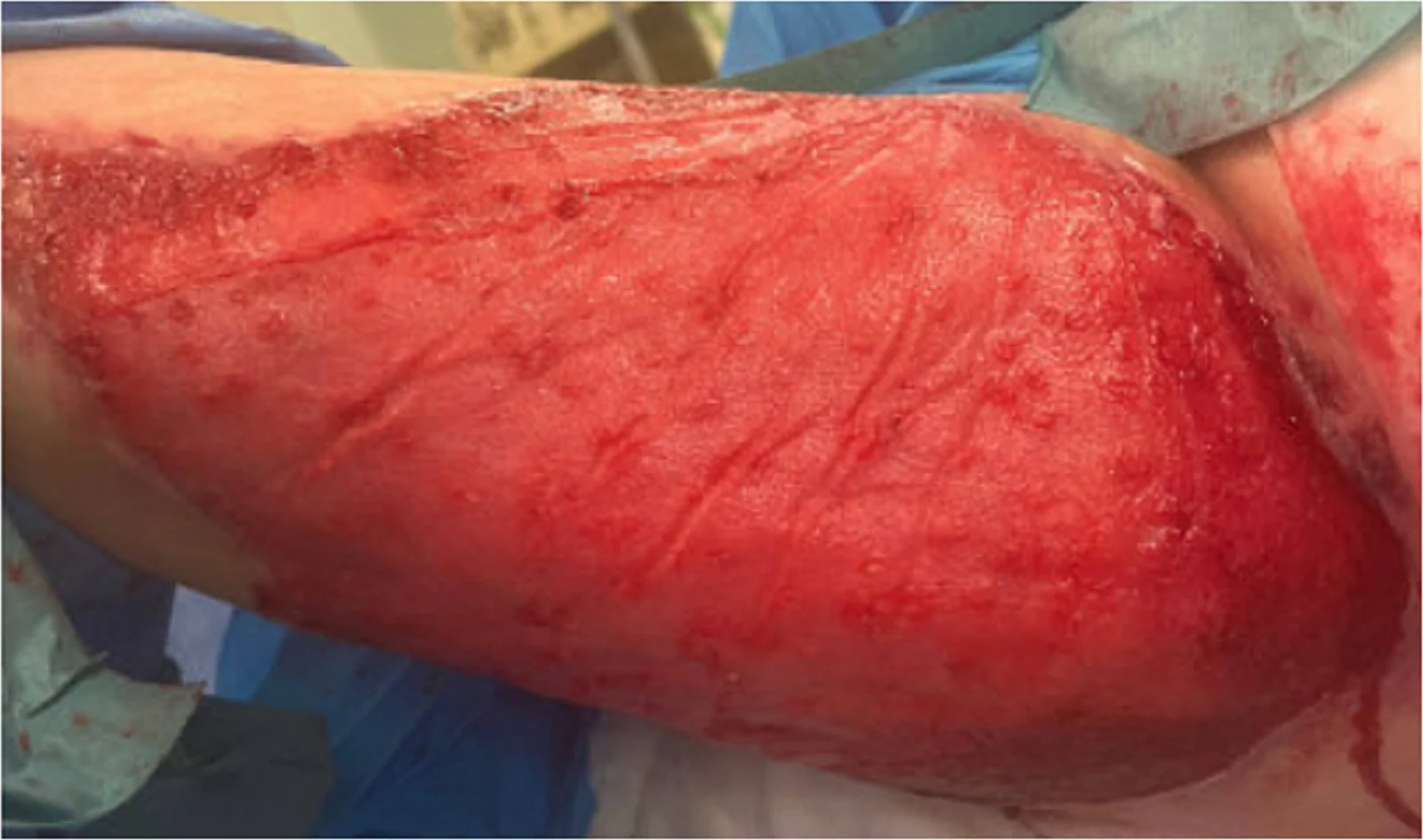
Intraoperative appearance at POD 44 showing 100% BTM take with well-vascularized blanching neo dermis prior to STSG application.

**Figure 5:**
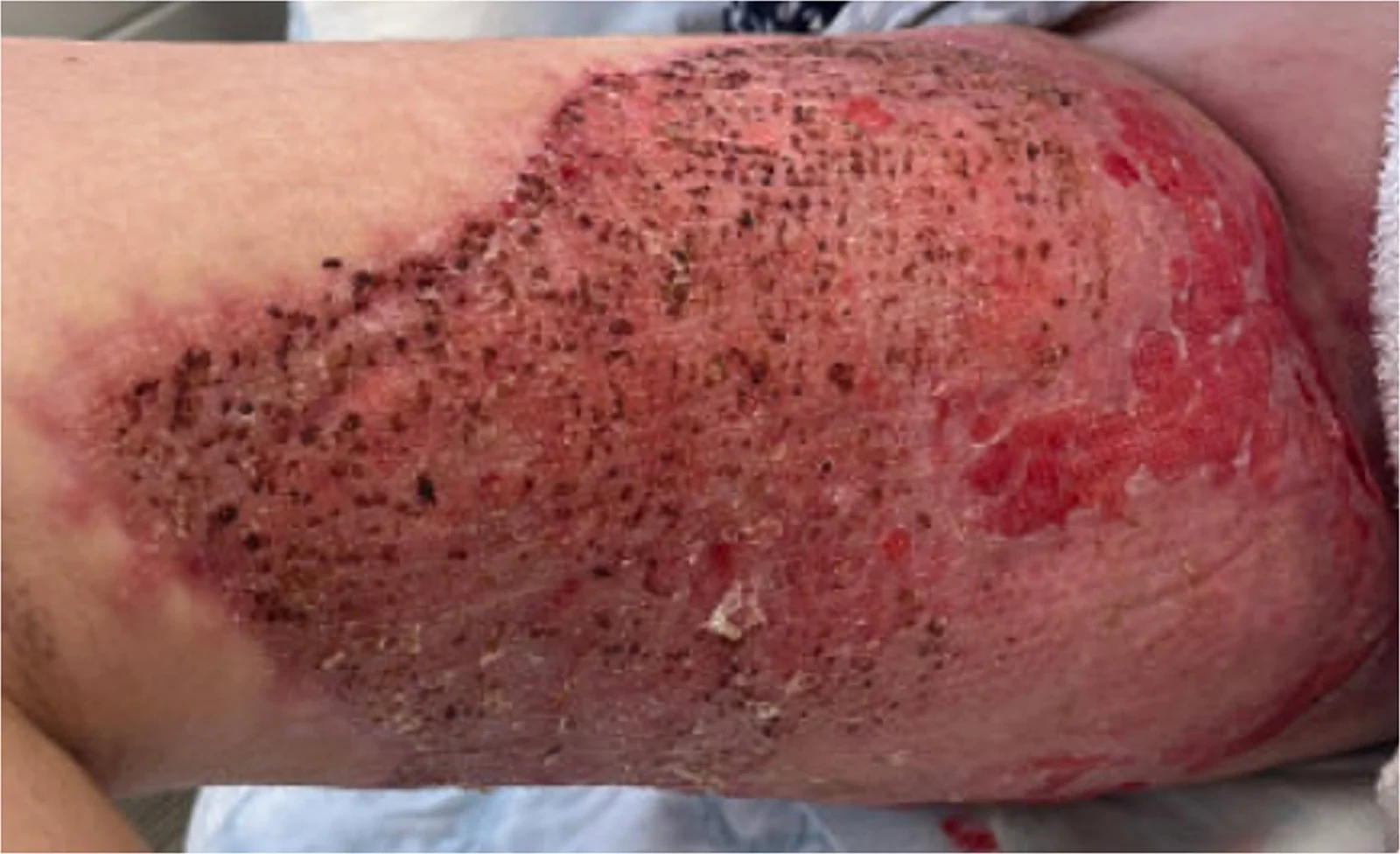
Medial thigh wound at POD 65 showing predominantly healed STSG with residual over-granulation at wound margins.

Total wound healing from excision took 4 months (Figs 6 and 7), by which time the patient had returned to school and social activities. At 1-year follow-up, the scar was fully healed and maturing, with possible early hypertrophic change along the graft seam (Figs 8–10). Scar optimization advice was given, including sun protection, regular moisturization, and scar massage. Procedures, wound status, and microbiology are further detailed in Table 1.

**Figure 6:**
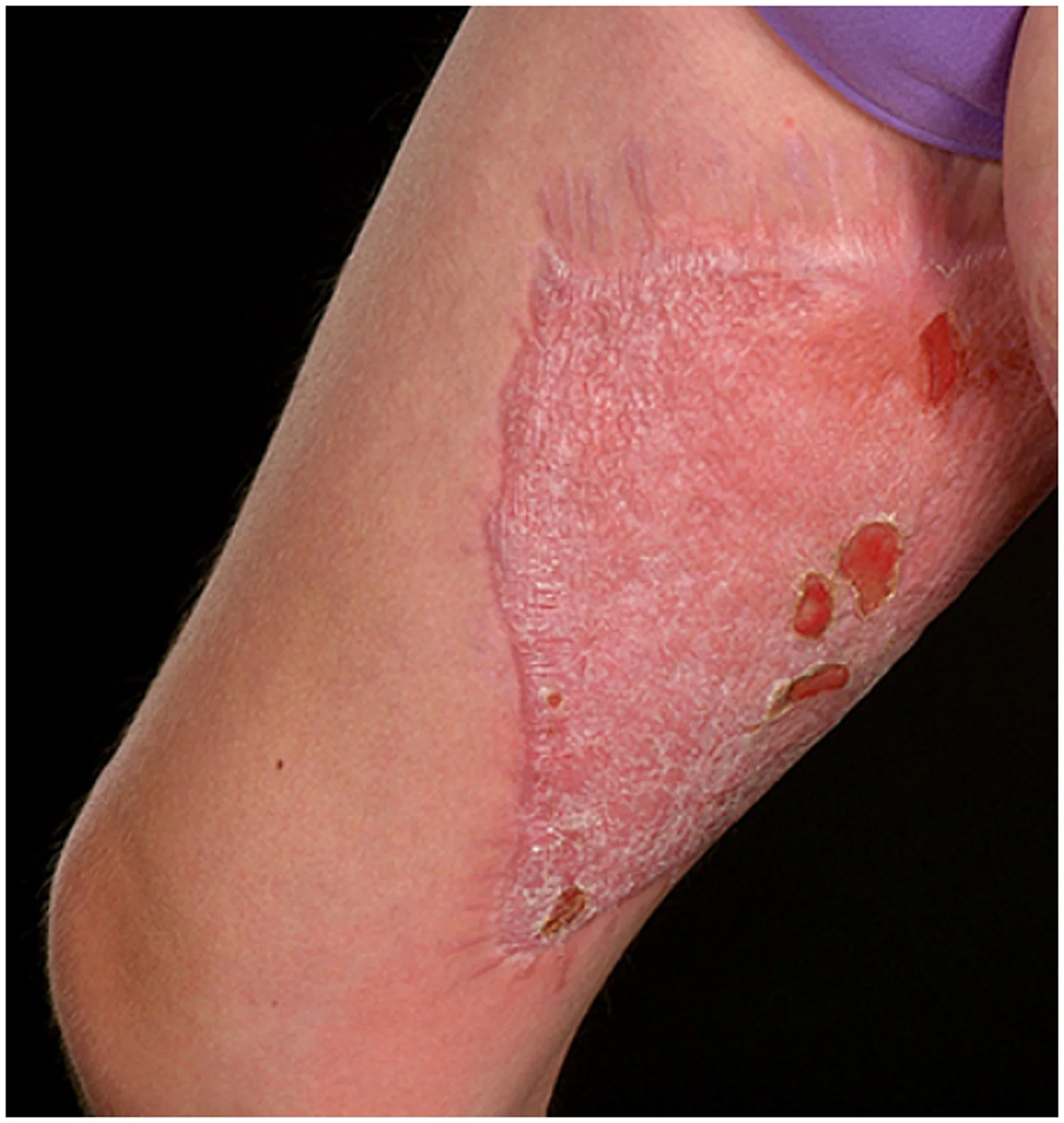
Medial thigh at 4 months showing largely healed wound with small residual epithelializing areas and good contour.

**Figure 7:**
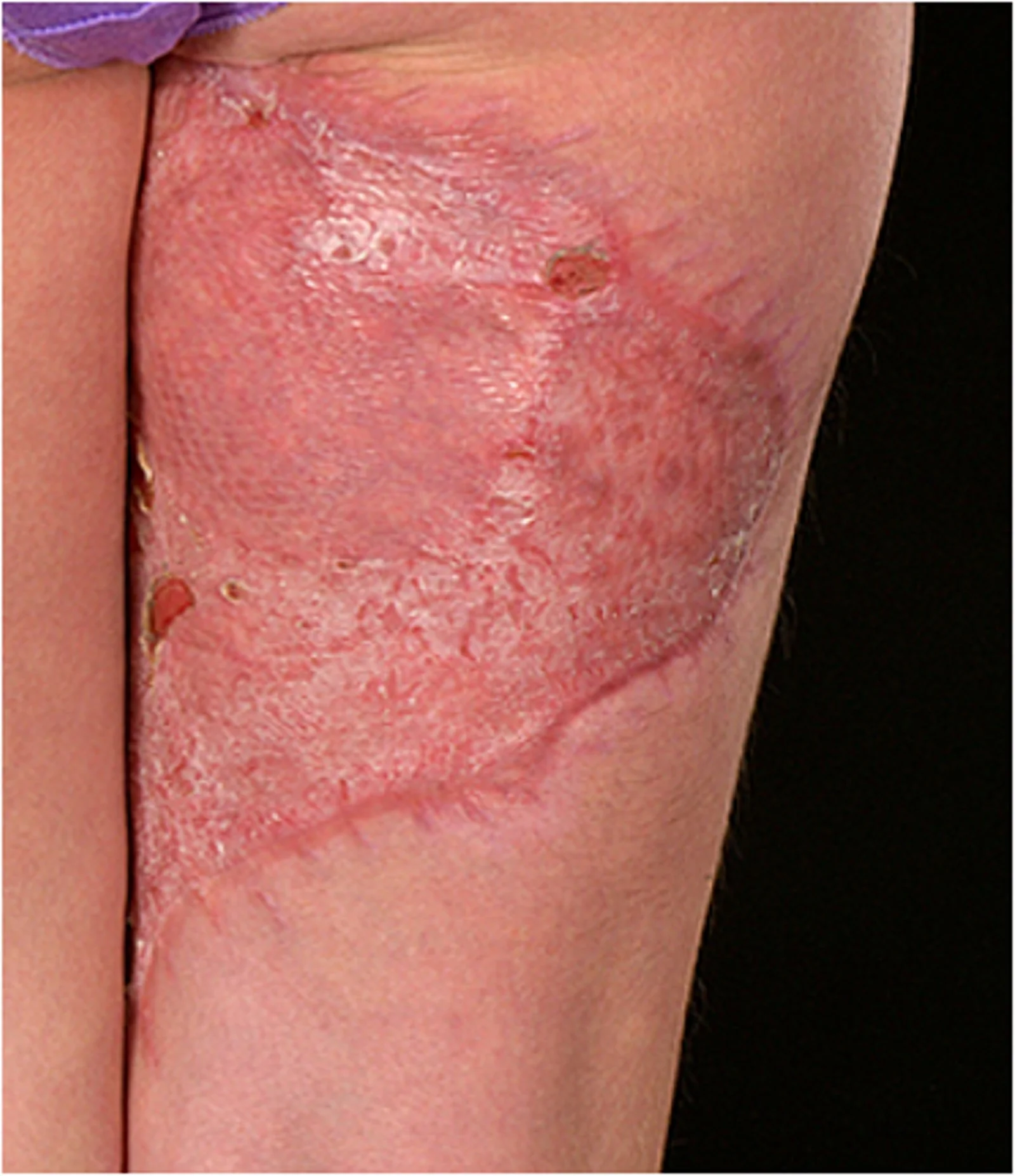
Medial thigh at 4 months showing lateral view of maturing scar with progressive colour fading.

**Figure 8:**
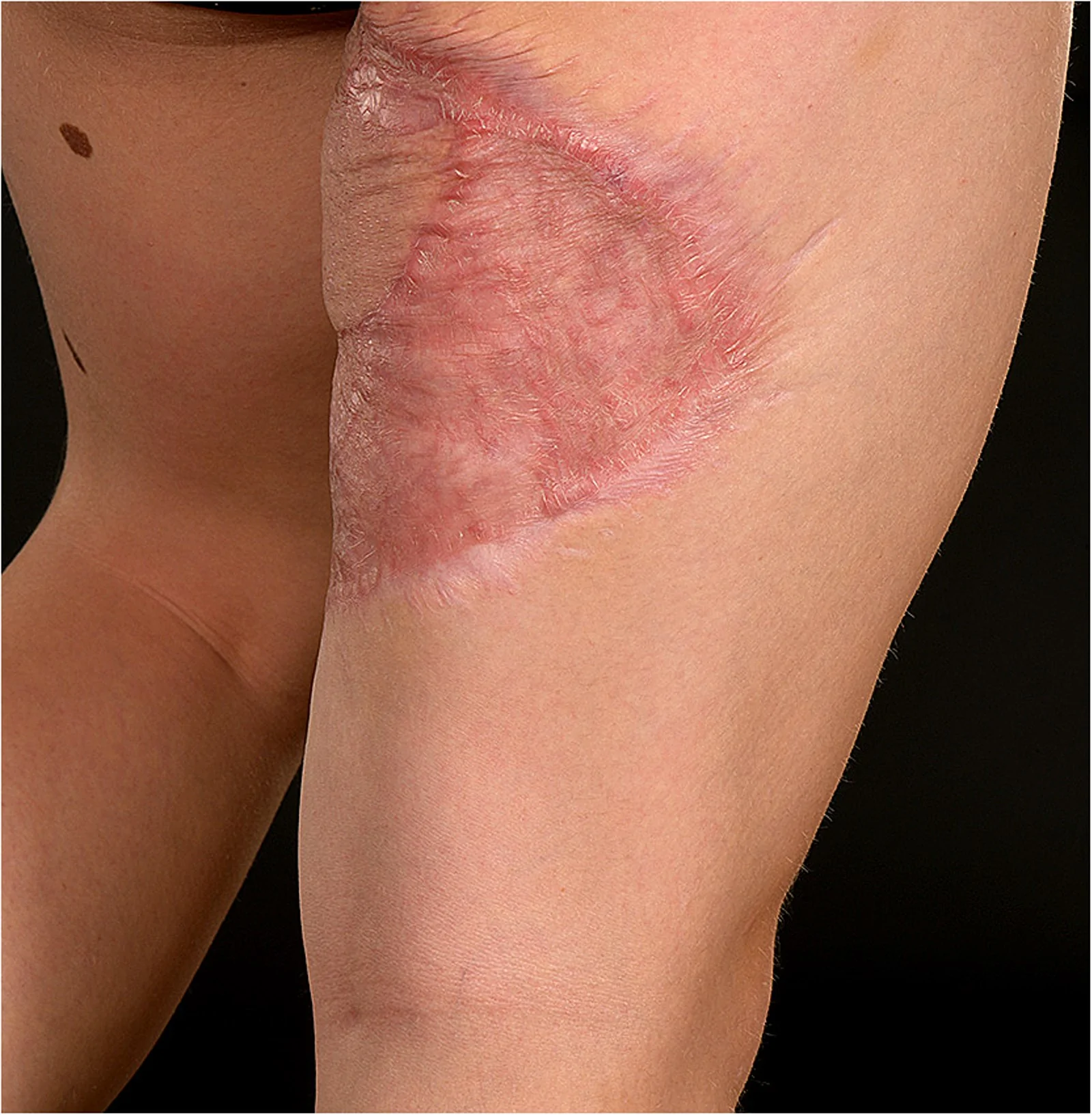
Right medial thigh at 1-year follow-up showing fully healed maturing scar with possible early hypertrophic change along the graft seam.

**Figure 9:**
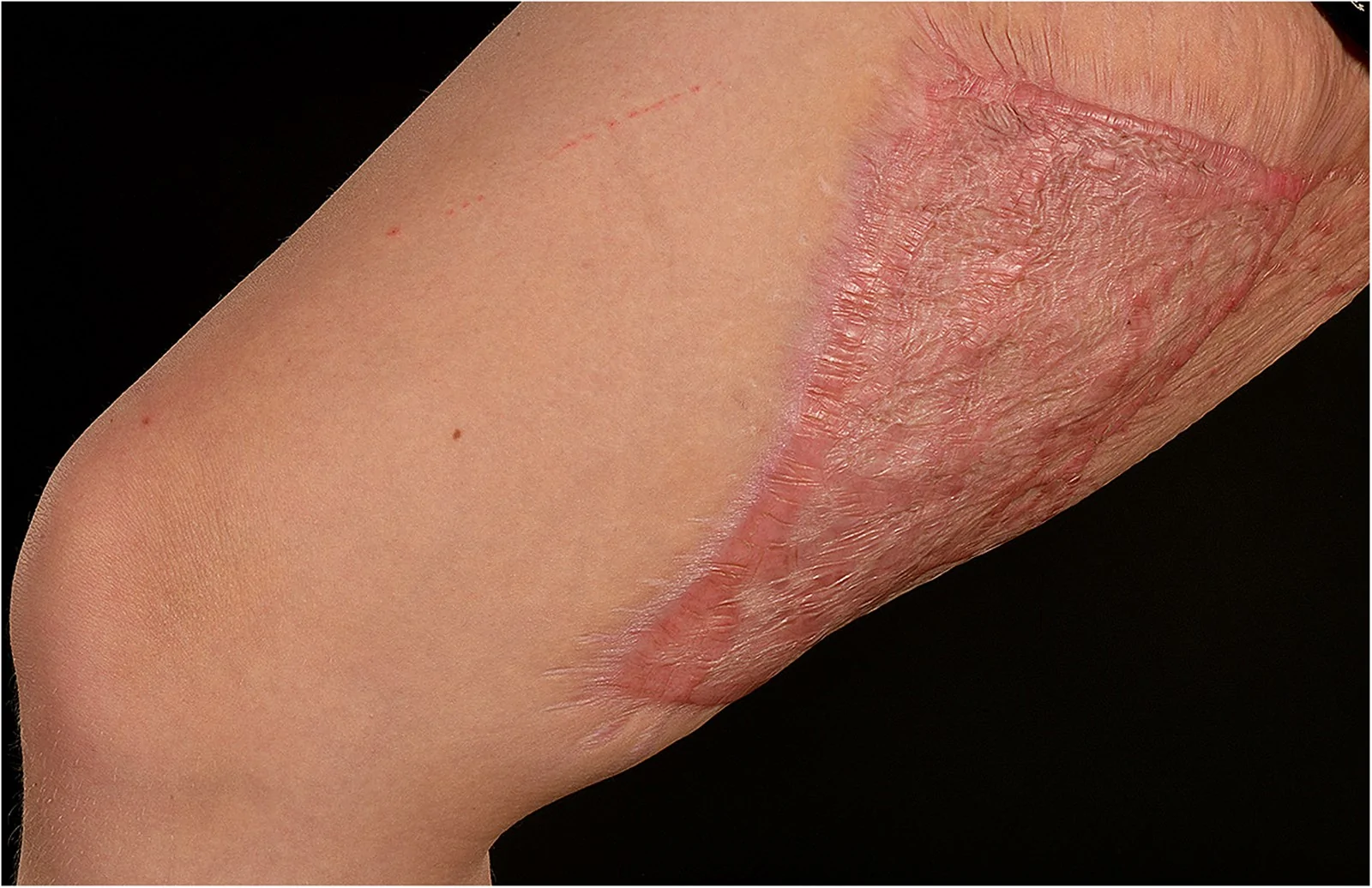
Right thigh at 1-year follow-up showing medial aspect of maturing scar with fading erythema.

**Figure 10:**
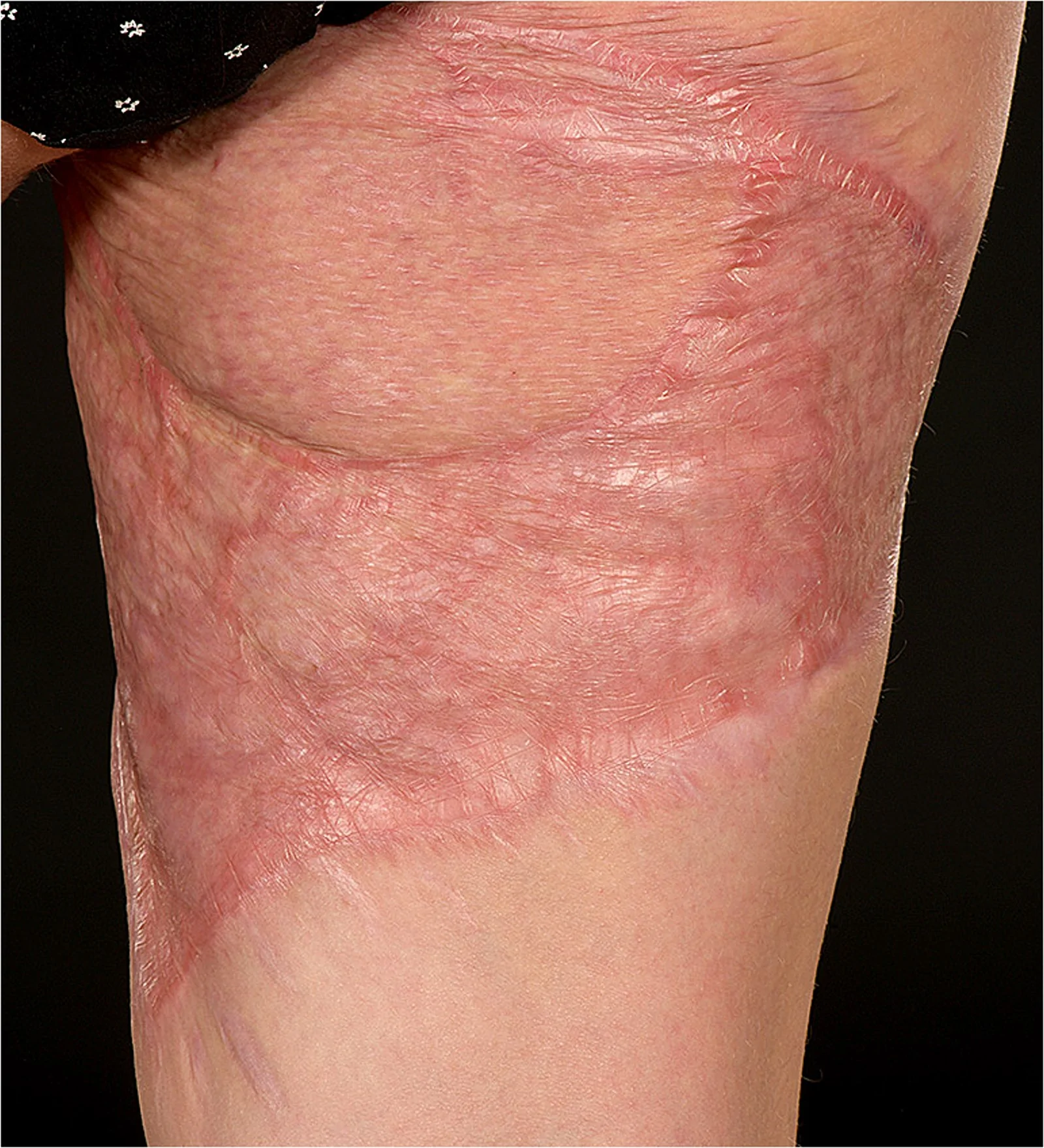
Right thigh at 1-year follow-up showing posterior aspect of fully healed maturing scar.

## Discussion

This case illustrates two clinically significant points: BTM’s capacity to withstand sustained infection and contamination in a high-risk location, and the importance of tailoring reconstruction boundaries to anatomical characteristics.

The decision to limit BTM application to the area proximal to the groin crease, with primary closure of the perineal extension, was deliberate and proved effective. Attempting BTM reconstruction into the perineum would have created a higher contamination risk and an unsealed VAC interface—both recognized factors for graft failure. These considerations for closure method are directly relevant for practitioners managing GCMN in the lower trunk or perineal regions.

The infection burden withstood during BTM integration was exceptional. This aligns with published data: from Solanki *et al*., e.g. reported a series in which BTM withstood *P. aeruginosa* contamination without requiring removal, with drainage and systemic antibiotics proving sufficient [8]. Prasidha *et al*. similarly documented BTM integration despite concurrent *Staphylococcus aureus* and *Pseudomonas aeruginosa* infection in a pyoderma gangrenosum wound without adverse tissue reaction [9]. In an anatomically comparable high-contamination setting, Gearing *et al*. reported successful BTM integration despite frank urine exposure following penoscrotal Paget disease reconstruction, with second-stage grafting achieved within 4 weeks [10].

The paediatric dimension adds further complexity. Storey *et al*.’s series of 63 paediatric complex wound cases found BTM reliably reduced wound contracture and provided the long-term flexibility required to accommodate patient growth [11]. A systematic review and meta-analysis by Lane *et al*. further supports BTM as effective in complex wound reconstruction across varied clinical contexts [12]. The two-stage application of BTM also offers a practical advantage over single-stage templates such as Matriderm®: should the STSG fail, the integrated dermal layer is not concurrently lost, preserving the option to re-graft without additional donor site harvesting [4, 13].

In conclusion, BTM demonstrated robust performance in a paediatric GCMN case complicated by serial infection, challenging anatomy, and a high-contamination wound environment. This case supports BTM as a viable option for complex paediatric wound reconstruction and highlights the importance of anatomically informed boundary decisions when planning dermal substitute application.
